# Tyrosine hydroxylase inhibits HCC progression by downregulating TGFβ/Smad signaling

**DOI:** 10.1186/s40001-024-01703-z

**Published:** 2024-04-12

**Authors:** Guoqian Liu, Mengwei Li, Zimei Zeng, Qi Fan, Xinxin Ren, Zhexin Wang, Yaoqi Sun, Yulin He, Lunquan Sun, Yuezhen Deng, Shupeng Liu, Chenxi Zhong, Jie Gao

**Affiliations:** 1 Key Laboratory of Molecular Radiation Oncology Hunan Province, Changsha, 410008 Hunan China; 2 Department of Thoracic Surgery, Shanghai Chest Hospital, Shanghai Jiao Tong University School of Medicine, Shanghai, 200030 China; 3 Cancer Center, Department of Pathology, Zhejiang Provincial People’s Hospital (Affiliated People’s Hospital), Hangzhou Medical College, Hangzhou, 310014 Zhejiang China; 4 Department of Obstetrics and Gynecology, Shanghai Tenth People’s Hospital, Tongji University School of Medicine, Shanghai, 200072 China; 5 Institute of Gynecological Minimally Invasive Medicine, Tongji University School of Medicine, Shanghai, 200072 China; 6 Xiangya Cancer Center, Xiangya Hospital, Central South University, 87th of Xiangya Road, Changsha, 410008 China

**Keywords:** Hepatocellular carcinoma, Cancer metabolism, Tyrosine hydroxylase, TGFβ/Smad signaling

## Abstract

The alteration of metabolic processes has been found to have significant impacts on the development of hepatocellular carcinoma (HCC). Nevertheless, the effects of dysfunction of tyrosine metabolism on the development of HCC remains to be discovered. This research demonstrated that tyrosine hydroxylase (TH), which responsible for the initial and limiting step in the bio-generation of the neuro-transmitters dopamine and adrenaline, et al. was shown to be reduced in HCC. Increased expression of TH was found facilitates the survival of HCC patients. In addition, decreased TH indicated larger tumor size, much more numbers of tumor, higher level of AFP, and the presence of cirrhosis. TH effectively impairs the growth and metastasis of HCC cells, a process dependent on the phosphorylation of serine residues (S19/S40). TH directly binds to Smad2 and hinders the cascade activation of TGFβ/Smad signaling with the treatment of TGFβ1. In summary, our study uncovered the non-metabolic functions of TH in the development of HCC and proposes that TH might be a promising biomarker for diagnosis as well as an innovative target for metastatic HCC.

**Supplementary Information:**

The online version contains supplementary material available at 10.1186/s40001-024-01703-z.

## Introduction

Liver cancer, predominantly hepatocellular carcinoma (HCC), is the 4th highest cancer-related mortality causes [1, 2]. HCC represents around 85–90% of all primary hepatic malignancies [3]. Curative surgery and loco-regional therapy are aggressive therapeutic treatments for individuals diagnosed with HCC [4]. Nevertheless, about 80% of HCC patients are unable to derive advantages from drastic therapies due to either local progression or distant metastases upon first diagnosis [5]. Despite significant advancements within the diagnosis and therapy of HCC, the overall outlook for long-term survival remains unfavorable [6]. Consequently, the identification of new biomarkers is imperative, which may be utilized for the diagnosis of HCC or targeted for therapy.

Tumor onset and progression are tightly influenced by deregulated metabolism [7]. Previous researches had identified the disruption of metabolism and the atypical expression of metabolic enzymes in HCC [8]. The integrated proteogenomic studies demonstrated that metabolic reprogramming in HBV-related HCC [9], namely involving PYCR2, ADH1A, et al. was associated with proteomic sub-grouping and played a role within the metabolic remodeling of HCC. The presence of mutated CTNNB1 was discovered to be associated with the phosphorylation of ALDOA, which in turn enhances glycolysis and cell proliferation. HCC cells have unique metabolic characteristics compared to normal hepatocytes, including significant alterations of the expression patterns of metabolic enzymes [10, 11]. The glucose transporters sodium–glucose cotransporter 2 (SGLT2) and glucose transporter 1 (GLUT1) shown abundantly increased expression in HCC and contribute to the development of HCC [12, 13]. Enzymes and substrates that involved in carbohydrate metabolism were found serve as specific pathological markers in the progression of HCC, consisting of those in glycolysis, gluconeogenesis, the pathway of pentose phosphate, as well as the tricarboxylic acid cycling (TCA cycling) [8]. Although metabolic enzymes play crucial roles in metabolic processes, there has been significant interest within their non-metabolic functions. Our previous researches have shown several kinases possess noteworthy non-metabolic roles. Nicotinamide adenine dinucleotide kinase plays a role in stabilizing the BMPR1A protein by binding with Smurf1. This interaction prevents the ubiquitination degradation of BMPR1A, leading to the initial cascade of BMP signaling in non-small-cell lung cancer. Consequently, this activation promotes the spread and planting of cancer cells to the lymph nodes [14]. Our additional research revealed that Nucleoside Diphosphate Kinase 7 interacts with and phosphorylates serine 9 of GSK3β, result in the upregulation of Wnt/β-catenin signaling, thus promotes one-carbon metabolism, ultimately contributes to the development of HCC [1].

Tyrosine hydroxylase, an enzyme of the non-heme iron and tetrahydrobiopterin (BH4)-dependent aromatic amino acid hydroxylase family, facilitates the transformation of L-tyrosine into L-3,4-dihydroxyphenylalanine. Limiting the initial reaction of biosynthesize catecholamines, such as dopamine, noradrenaline, as well as adrenaline [15]. Multiple serine residues have been reported to be phosphorylated [16], with phosphorylation of S19 and S40 being particularly important for sustaining TH activity and its conformational stability [15, 16]. Research has demonstrated that an increased expression of TH was a positive prediction for the OS as well as event-free survival of patients suffering from neuroblastoma [17]. Nevertheless, the function of TH within the HCC development remains ambiguous.

The dysregulated TGFβ/Smad signaling is significantly involved in inflammation, fibro-genesis, and particularly metastasis of HCC [18]. Initial activation of TGFβ/Smad pathway through specific TGFβ ligand binding to its membrane receptors, following result in the phosphorylation of Smad2/3 and the increased transcriptional expression of downstream target genes, such as fibronectin and CTGF. Atypical activation of TGFβ/Smad pathway enhanced the migratory capacity of cancer cells by initiating the process of EMT [19].

This research investigated the expression pattern, bio-functions, and molecular mechanisms of TH in the development of HCC.

## Materials and methods

### Cell culture and clinical hepatocellular carcinoma samples

The HHL5 normal human hepatic cells and HCC cell lines (Huh7, QGY7701, MHCC97H, QGY, as well as 7404) were procured from the Cell Bank of the Chinese Academy of Sciences in Shanghai, China. The HCC cell line PVTT was acquired from Shanghai Institute of Nutrition and Health, which is part of the Chinese Academy of Sciences. As for culture medium, 7404 cells were cultivated in RPMI-1640 medium supplied with 10% FBS, whereas remainder group of cells were cultured in DMEM medium supplied with 10% FBS. Both medium contained penicillin and streptomycin with a concentration of 100 U/mL, 100 μg/mL, respectively. In addition, the cultivation environment was sterile incubator at a temperature of 37 ℃ containing 5% CO_2_.

Hepatocellular carcinoma samples and adjacent non-tumor tissues were acquired from Shanghai Eastern Hepatobiliary Surgery Hospital, affiliated to the Second Military Medical University, after patient consent. This cohort consists of 65 HCC tissues and corresponding adjacent non-tumor tissues, which were used to evaluating the mRNA levels of tyrosine hydroxylase. The study received the endorsement of the Ethics Committee of the Second Military Medical University for all experimental procedures.

### Plasmids and transfection

To induce TH overexpression, its coding sequence was cloned into the pLVX–IRES-puro vector. Sequences targeting TH for RNA interference were selected out of the Merck site (https://www.sigmaaldrich.cn/CN/zh/product/sigma/shrna) and inserted to the pLKO.1-puro vector. These targeted shRNA sequences included:

shTH #1 F: 5ʹ-CCGGGGTGTTTGAGACGTTTGAAGCCTCGAGGCTTCAAACGTCTCAAACACCTTTTTG-3ʹ,

shTH #1 R: 5ʹ-AATTCAAAAAGGTGTTTGAGACGTTTGAAGCCTCGAGGCTTCAAACGTCTCAAACACC-3ʹ;

shTH #2 F: 5ʹ-CGGGCTGGACAAGTGTCATCACCTCTCGAGAGGTGATGACACTTGTCCAGCTTTTTG-3ʹ,

shTH #2 R: 5ʹ-AATTCAAAAAGCTGGACAAGTGTCATCACCTCTCGAGAGGTGATGACACTTGTCCAGC-3ʹ.

The Smad2 coding sequence was inserted into the pGEX-4T1 vector to stimulate the production of a fusion protein called GST–Smad2 in prokaryotic cells with IPTG.

The psPAX2 and pMD2.G, served as lentiviral packaging plasmids, were co-transfected with plasmids inserted into specific sequences into HEK293T cells by employing lipofectamine 8000 transfection Reagent. After a period of 24 h, The liquid portion of the mixture was gathered at a period of 48 h and 72 h after transfection. PEG8000 was introduced to this liquid portion, and it was subjected to centrifugation for 1 h at a temperature of 4 ℃ and a centrifugal pull of 1600 × *g*. This process was carried out to cleanse the lentivirus. Following the centrifugation process, the liquid portion above the sediment was removed, and the concentrated viral was overnight dissolved in 2 mL of basal medium. Next, 4 × 10^5^ cells were infected with 400 μL of lentivirus solution. Following a 24 h incubation, the cells were passaged and subjected to puromycin screening with a concentration of 2 μg/mL. Expression of the target protein was subsequently measured by Western blot analysis.

### Extraction of RNA with a quantitative polymerase chain reaction

The RNA was isolated using TRIzol and specific mass of RNA was converted into complementary DNA by using the PrimeScript RT reagent kit (Takara). The qPCR examination was conducted using the SYBR Green Kit and the CFX96 real-time fluorescence qPCR system from BIO-RAD. Actin was utilized as an internal control. The 2^−ΔΔCt^ of target genes were calculated. The primer sequences carried out for TH in this section are the following:

TH F: 5ʹ-CATGGTAAGAGGGCAGGGC-3ʹ,

TH R: 5ʹ-GTGTAGGATGCAGCTGGGG-3ʹ.

### Western blot

The PBS buffer was used for the purpose of washing the cells. And then, cells were lysed on ice by using RIPA lysis buffer containing protease inhibitors along with phosphatase inhibitors. The cellular extract was collected and subjected to centrifugation at 4 ℃ and 14,000 rpm for a duration of 20 min. The protein concentration was measured with the BCA kit. The protein samples were combined with protein loading buffer and subjected to heat at 100 ℃ for a duration of 5 min. The protein was transfer printed to a polyvinylidene difluoride membrane and then incubated with a 5% bovine serum albumin solution to block nonspecific binding. Subsequently, the membrane was subjected to incubation with targeted primary antibodies at a temperature of 4 ℃ for the duration of 8–10 h. On the following day, the membrane was cleansed employing TBST and then exposed to HRP-coupled secondary antibodies for a duration of 1–2 h. The membrane was cleansed with TBST, the signals were identified employing a chemiluminescent reagent (Millipore, WBKLS0050), and protein bands were examined employing Image Lab (BIO-RAD).

The antibodies utilized for such investigation were listed: TH (Brand &Cat no: Proteintech, 25859-1-AP; Dilution: 1:2000), Flag-tag (Brand &Cat no: Proteintech, 20543-1-AP; Dilution: 1:3000), HA-tag (Brand &Cat no: Proteintech, 66006-Ig; Dilution: 1:3000), GST-tag (Brand &Cat no: Proteintech, 10000-0-AP; Dilution: 1:3000), β-Tubulin (Brand &Cat no: Proteintech, 10068-1-AP; Dilution: 1:3000), GAPDH (Brand &Cat no: Proteintech, 10494-1-AP; Dilution: 1:10,000), Actin (Brand &Cat no: Santa Cruz, SC-8432; Dilution: 1:3000), HSP90 (Brand &Cat no: Proteintech, 13171-1-AP; Dilution: 1:3000), Smad2 (Brand &Cat no: Proteintech, 12570-1-AP; Dilution: 1:3000), and phospho-Smad2 (ser465/ser467) (Brand &Cat no: CST, 18338S; Dilution: 1:1000).

### Immunohistochemistry

The dewaxed tissue sections were immersed in an EDTA solution and subjected to boiling at a temperature of 100 ℃ for a duration of 30 min to facilitate the recovery of antigens. Once the tissues reached room temperature through ambient cooling. The endogenous peroxidase activity was inhibited by employing endogenous peroxidase blockers. Subsequently, the sections underwent three rounds of washing in PBS, followed by incubation with three percent normal goat serum for a duration of 30 min at room temperature. The TH antibody (Brand &Cat no: Abcam, EP1532Y; Dilution: 1:500) was incubated with the sections overnight at a temperature of 4 ℃. The next day, the sections were washed three times with PBS and then incubated with secondary antibodies for 1 h at 37 ℃. Immunohistochemical staining was conducted utilizing 3,3ʹ-diaminobenzidine, while the nuclei were stained with hematoxylin. The Inform 2.4.0 system, developed by PerkinElmer, was utilized to assess the H-scores of immunohistochemical staining. The Kaplan–Meier technique was utilized to draw survival curves, and survival analysis was conducted employing the log-rank test.

### Immunoprecipitation

To identify the binding between externally introduced TH and Smad2, the Flag-TH plasmid and the HA–Smad2 plasmid were simultaneously introduced into HEK293T cells employing transfection. After 48 h of transfection, the HEK293T cells were lysed employing IP lysis buffer, including protease inhibitors and phosphatase inhibitors. The IP lysis buffer composition included a solution containing 50 mM Tris–HCl (pH 7.4), 150 mM NaCl, and 5 mM EDTA, and 1% NP-40. The supernatants were collected after centrifugation at 4 ℃ and 14,000 rpm for 20 min. Add Flag-beads (Brand &Cat no: Sigma, A2220) or HA-beads (Brand &Cat no: Thermo Fisher, 88837) to cell supernatants incubate in rotate at a temperature of 4 ℃ for a duration of 3 h. The beads underwent three rounds of washing with wash buffer. Subsequently, the beads were mixed with protein loading buffer and subjected to heating at a temperature of 100 ℃ for a duration of 5 min. Western blot analysis was following conducted to detect the interaction.

To confirm the binding between endogenous TH and endogenous Smad2 in liver cancer cells, Huh7 cells were lysed employing an IP lysis buffer contains protease inhibitors and phosphatase inhibitors. Cell supernatants were following have an incubation with 2 μg of anti-TH antibody or 2 μg of anti-Smad2 antibody at a temperature of 4 ℃ for the duration of 8 h. The next day, 25 μL Protein A/G beads (Brand &Cat no: Bimake, B23202) were rotated incubated with the supernatant at a temperature of 4 ℃ for a duration of 2 h. The beads underwent three rounds of washing with IP lysis buffer. Samples were mixed with protein loading buffer, heated at 100 ℃ for 5 min, and then subjected to Western blot.

### GST-pull down

The induction of GST as well as GST–Smad2 fusion protein expression in BL21 codon competence cells was achieved by employing IPTG (Brand &Cat no: Sangon, A100487) at a dose of 1 μM. The bacteria precipitations were subsequently lysed employing an IP lysis solution supplemented with protease and phosphatase inhibitors. Above lysate was subjected into incubation with GST beads at a temperature of 4 ℃ for a duration of 2 h.

The Flag-TH plasmids were introduced into HEK293T cells by transfection, and after 48 h, the cells were lysed employing an IP lysis solution. Enriched GST–Smad2 proteins and GST coupling with GST beads, were incubated with the cell lysate supernatant for 3 h at 4 ℃. Following three times of washes with IP wish buffer, the beads were mixed with 50 μL protein loading buffer and denatured at 100 ℃ for 5 min. The protein directly binding involving TH and Smad2 was examined employing Western blot assessment.

### In vivo metastasis assay

This experiment utilized BALB/c-Nude mice (Brand: Gempharmatech), specifically male mice aged 5–6 weeks. Huh7 cells were subjected to lentivirus-mediated luciferase expression and subsequently screened upon treatment of Hygromycin B. Subsequently, each nude mice received an injection of 1 × 10^6^ cells through the tail vein. Following a duration of 4 weeks, the nude mice were administered an intraperitoneal injection of 3 mg of D-fluorescein potassium salt (Brand &Cat no: Beyotime, ST196) and subsequently sedated with isoflurane. After a duration of 10 min, the bioluminescence of the mice was examined utilizing small animal bioluminescence imaging.

### Soft agar assay

Upon reaching a cell confluence of 70–80%, the cells underwent digestion, and cells were resuspended in basal medium. The bottom gel with a volume of 300 μL per well, consisting of 20% FBS, 40% 2 × MEM, and 40% 1.25% agar. The culture layer with a volume of 400 μL per well, with a mixture of 25% FBS, 37.5% 2 × MEM, 37.5% 1% agar, 0.8% 2 mM L-glutamine and 800 cells. This assay was performed on 24-well plates, with PBS surrounded to sustain a moist culture environment. There were 4 replicate wells per cell. The colony was cultured at a temperature of 37 ℃ with a 5% concentration of carbon dioxide for a duration of 10–14 days. Five samples were chosen at random from per well for cell colony counting employing a microscope.

### Transwell invasion assay

Growth factor-free Matrigel was diluted with basal DMEM medium at a ratio of 3:100, gently mixed, and promptly applied to the surface of the upper compartment. After 30 min, the liquid portion was removed. The lower chamber was replaced with 500 μL of DMEM medium with 30% FBS. The cells were resuspended with basic DMEM media at a concentration of 1 × 10^5^ cells per milliliter. Following the mixing process, 200 μL cell solution containing 2 × 10^4^ cells was placed within the upper chamber. After incubated at 37 ℃ with 5% CO_2_ for a duration of 48 h. The cells were fixed with a 4% paraformaldehyde solution and then subjected to staining with crystal violet. Five fields were chosen from each transwell chamber, and photographs were captured employing a microscope.

### CCK8 assay

Every well of 96-well plates was seeded with 1 × 10^3^ cells and then cultivated within the incubator at a temperature of 37 ℃ with a CO_2_ concentration of 5%. The following 5 days, the fresh basic medium with a concentration of 10% Cell Counting Kit-8 solution was added into the wells after using the negative pressure aspirator to suck the old medium away. After a 2-h incubation, the optical density at 450 nm was measured. Proliferation rate was drawn by a curve of OD450.

### Wound healing assay

The HCC cell lines were distributed into culture dishes with a diameter of 3.5 cm with the same number of cells. Upon reaching full cell confluence, a linear wounding was created employing a pipette tip. Healing progression of wound was subsequently recorded employing microscopy at various time intervals. The assessment of wound healing rate of each cell was conducted using the Image J program.

### Statistical analysis

The experiment results were analyzed by using SPSS Version 23.0 and GraphPad Prism Version 8.0.2 (GraphPad software, La Jolla, CA, US). Data were assessed using log-rank tests, two-sided χ2 tests, and two-tailed Student's *t* tests. The experiment results were considered to be statistically significant when the *p* value was less than 0.05.

## Results

### Decreased TH expression in HCC tissues facilitates HCC patient survival

**Fig. 1:**
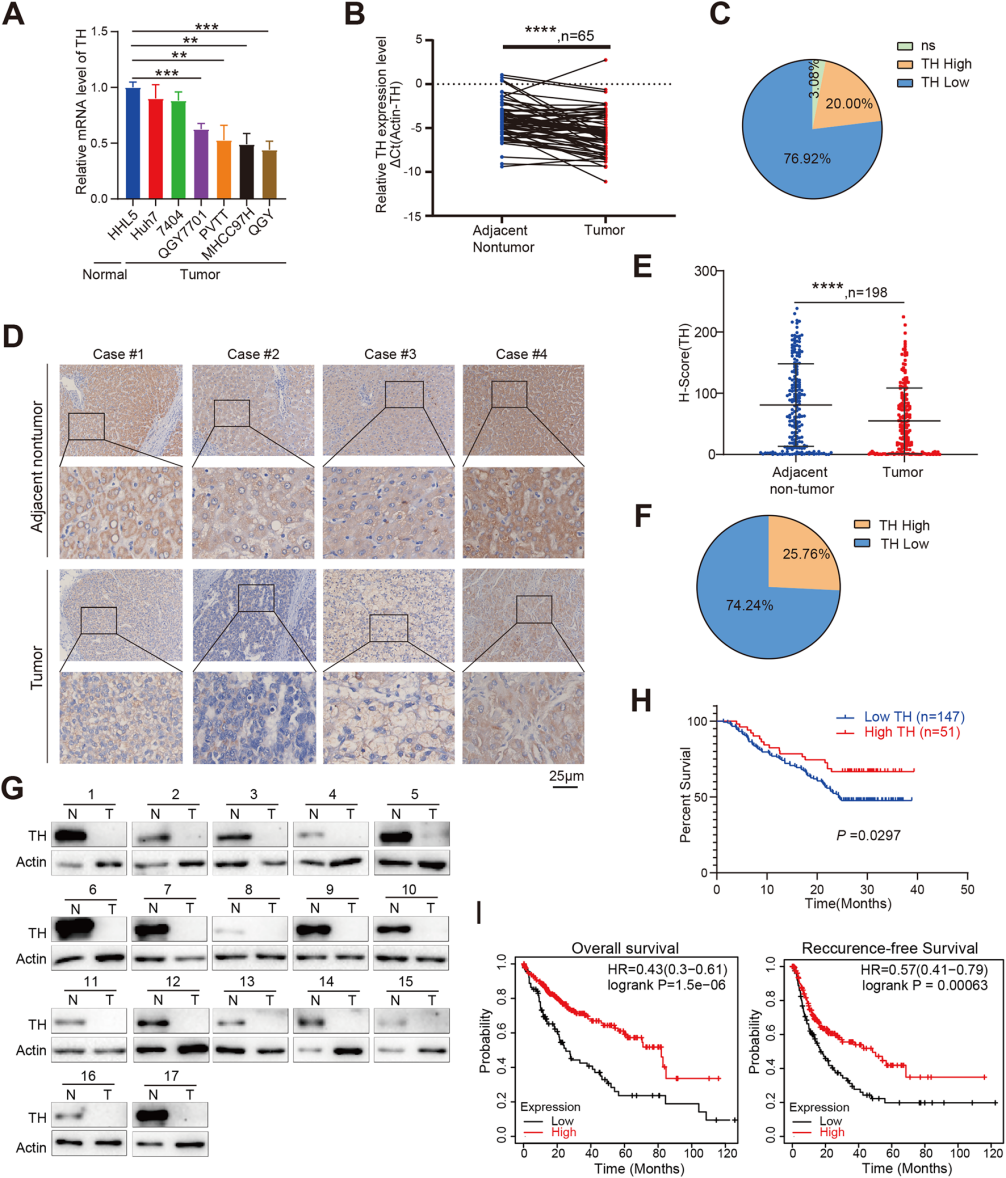
Expression of TH in HCC tissues was down-regulated. **A** Relative mRNA levels of TH in normal hepatic cells ((HHL5) and HCC cell lines (Huh7, 7404, QGY7701, PVTT, MHCC97H, and QGY) was quantified by qPCR, normal hepatic cells ((HHL5) acts as the statistical comparison. **P < 0.01; ***P < 0.001. **B** qPCR was used to quantify the TH mRNA levels in 65 HCC tissues and adjacent normal tissues. Paired *t* test was used to analyze. ****P < 0.0001. **C** Pie chart illustrates the comparative mRNA levels in 65 HCC tissues as compared to their corresponding adjacent non-tumor tissues. “Low”, lower TH mRNA level in tumor; “High”, higher TH mRNA level in tumor; “NS”, no significance. **D**, **E** Immunohistochemistry was used to detect the protein expression of TH in tumor tissues and adjacent non-tumor tissues (**D**), quantified H-score for TH expression in 198 HCC tissues and relative adjacent non-tumor tissues was shown (**E**). ****P < 0.0001. **F** Pie chart displays the proportion of HCC tissues that have either higher or lower TH protein levels in comparison to the corresponding non-tumor tissues. **G** Western blotting was used to detect the protein level of TH in 17 HCC samples and their matched non-tumor tissues. **H** Conducting an analysis to reveal the correlation between the protein expression of TH and the overall survival of 198 patients diagnosed with HCC. **I** Kaplan–Meier Plotter database was utilized to analyze the relevance between TH mRNA expression and overall and recurrence-free survival in HCC patients

Initially, we quantified the expression level of TH mRNA and protein in HCC cells and tissues. The mRNA expression level of TH in the majority of hepatocellular carcinoma cell lines, including QGY7701, PVTT, MHCC97H, and QGY, was shown to be lower compared to the normal hepatic cell line, HHL5 (Fig. 1A). The measurement of mRNA expression levels in 65 HCC tissues and matched non-tumor tissues revealed a decrease in TH expression in tumor tissue compared to the adjacent non-tumor tissues (Fig. 1B). In addition, the mRNA level of TH was found to be down-regulated in nearly three quarters (76.92%) of HCC tissues (Fig. 1C). Subsequently, immunohistochemistry (IHC) was utilized to quantify the levels of TH protein expression in a human HCC tissue array consisting of 198 HCC samples together with their corresponding adjacent non-tumor tissues. The protein expression level of TH in the HCC tissues was found to be lower than that in the adjacent non-tumor tissues (Fig. 1D and E). Similarly, the TH protein level was lower in nearly three quarters (74.24%) of the HCC tissue samples in contrast with the adjacent non-tumor tissue (Fig. 1F). The protein expression level of TH measured by western blot consistent with IHC assay, 17/17 of tumor tissues shown down-regulated compare with adjacent non-tumor haptic tissues (Fig. 1G). Furthermore, negative correlation was seen between the levels of TH protein and the tumor size, tumor number, the level of AFP, as well as cirrhosis (Table 1). The survival analysis demonstrated that the decreased TH expression in tumor tissues was associated with worse survival outcomes in individuals with HCC (Fig. 1H). Furthermore, overall survival and recurrence-free survival analysis employing the Kaplan–Meier plotter database uncovered the positive correlation between the mRNA level of TH and the survival of patients (Fig. 1I). These data indicate that the decrease in TH expression in HCC may enhance the development of HCC.

**Table 1:** Relevance between TH protein expression and the clinical characteristics of individuals with hepatocellular carcinoma

Characteristic	Total	TH Expression		χ^2^	*P*
		Low (n=147)	High (n=51)		
Age					
≥55	182	135	47	0.005	0.604
<55	16	12	4		
Gender					
Male	68	59	9	8.493	0.002
Female	130	88	42		
Tumor size					
≥11	38	33	5	3.904	0.034*
<11	160	114	33		
Tumor number					
≥2	24	22	2	4.336	0.026*
≤1	174	125	49		
PVTT					
Yes	26	18	8	0.393	0.341
No	172	129	43		
Tumor grade					
I–II	48	35	13	2.898	0.235
III	150	112	38		
HBV infection					
Yes	88	61	27	2.009	0.105
No	110	86	24		
AFP(μg/L)					
≥400	84	69	15	6.291	0.043*
25~400	67	35	12		
≤25	47	43	24		
Paracancerous microtumor					
Yes	70	49	21	1.019	0.2
No	128	98	30		
Cirrhosis					
Yes	186	141	45	3.926	0.048*
No	12	6	6		
Vascular tumor thrombus in cancer nest					
Yes	84	63	21	0.044	0.484
No	114	84	30		
Tumor capsular					
Yes	115	86	29	0.042	0.482
No	83	61	22		

### TH suppresses the migration, proliferation, and anchor-independent growth of HCC cells

**Fig. 2:**
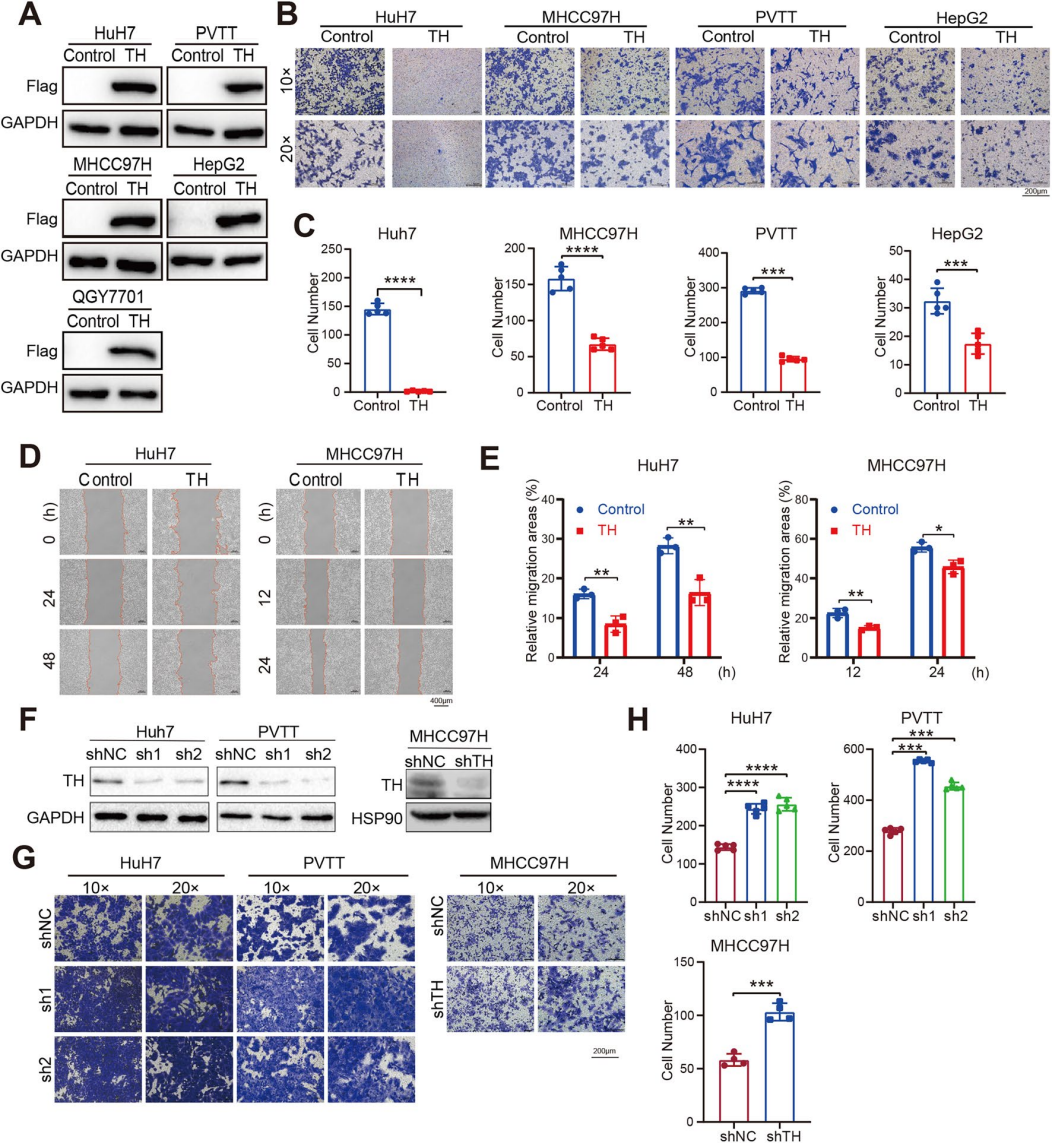
TH suppresses the migration and invasion of HCC cells. **A** Western Blotting was utilized to detect the overexpression of Flag-TH in HCC cell lines, including Huh7, PVTT, MHCC97H, HepG2, and QGY7701. **B**, **C** Transwell invasion assays (**B**, **C**) was conducted to measure the impact of TH overexpression on the invasion ability of HCC cells (**B**). These invasion cells were counted and analyzed (**C**). **P < 0.01; ****P < 0.0001. **D**, **E** Impact of TH overexpression on the migration of HCC cells was evaluated employing a wound-healing experiment (**D**). The relative healing areas were quantified and analyzed (**E**) ***P < 0.001; ****P < 0.0001. **F** Western blotting was conducted to measure the interfere impact of shRNA on the expression of TH protein in HCC cells. **G**, **H** Transwell invasion assays were applied to investigate the impact of suppressing TH on the invasion ability of Huh7, PVTT, as well as MHCC97H cell lines (**G**). The number of invasive cells was then counted and analyzed (**H**). The presence of scale bars was highlighted. ***P < 0.001; ****P < 0.0001

To uncover the biology functions of TH in the development of HCC, we established five HCC cell lines overexpressing Flag-TH (Huh7, PVTT, MHCC97H, HepG2, and QGY7701) (Fig. 2A). Transwell invasion assays demonstrated that the number of invasion cells were reduced in HCC cells overexpressing Flag-TH (Fig. 2B, C). In addition, the wound-healing assay demonstrated that the overexpression of TH suppressed the migration of HCC cells (Fig. 2D, E). To further confirm the inhibitory function of TH in the development of HCC, we used two distinct short hairpin RNAs (shRNAs) to knock down the expression of TH in Huh7 and PVTT cell lines. In addition, we carried out an efficient shRNA to knock down TH expression in MHCC97H cell lines (Fig. 2F). The result of transwell invasion assay demonstrated that the interfere of TH facilitated the invasion of HCC cells (Fig. 2G, H). To summarize, TH suppressed the migration and invasion of HCC cells.

**Fig. 3:**
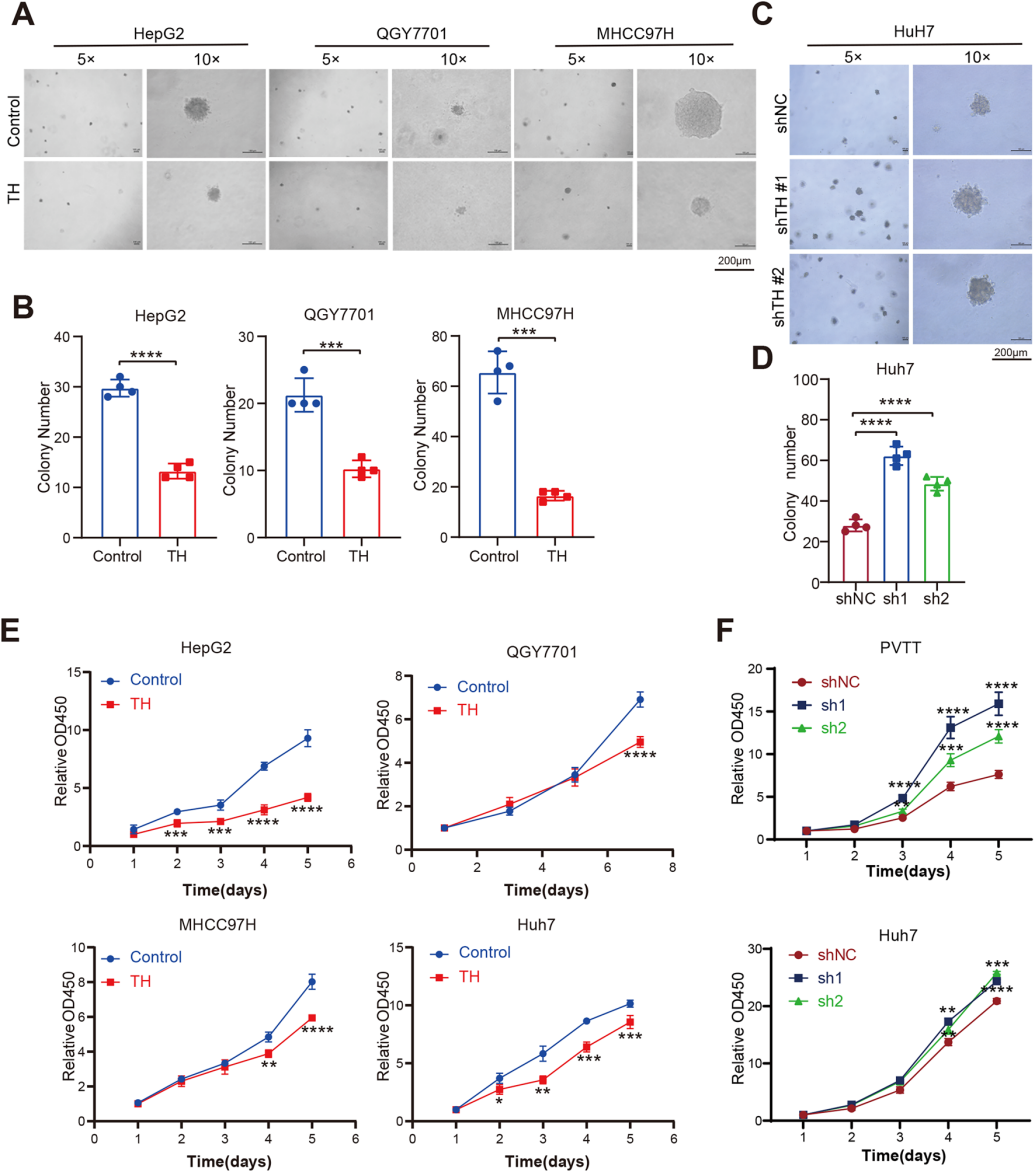
TH suppresses the proliferation and anchor-independent growth of HCC cells. **A**, **B** Impact of TH overexpression on the anchor-independent growth ability of HCC cells (HepG2, QGY7701, and MHCC97H) was evaluated by applying the soft-agar assays (**A**). The colony number was counted and analyzed (**B**). ***P < 0.001. **C**, **D** Soft-agar assays was conducted to examine the impact of TH knockdown on the anchor-independent growth ability of Huh7 cell lines (**C**). The number of colonies were counted and analyzed (**D**). The statistical significance levels are as follows: ****P < 0.0001. **E** Proliferation ability of HCC cells with TH overexpression was measured using the CCK8 assays. The presence of scale bars was highlighted. *P < 0.05; **P < 0.01; ***P < 0.001; ****P < 0.0001. **F** The CCK8 assay was conducted to test the proliferation rate of HCC cells with TH knock-down. **P < 0.01; ***P < 0.001; ****P < 0.0001

In addition, we investigated the impact of TH on HCC cells proliferation. The soft-agar colony formation experiment revealed that overexpression of TH suppressed the anchorage-independence growth ability of HCC cell lines (HepG2, QGY7701, and MHCC97H) (Fig. 3A, B). Nevertheless, the suppression of TH in HCC cells facilitated anchorage-independent growth (Fig. 3C, D). Furthermore, the cell proliferation ability in liquid culture of HCC cell lines were suppressed after overexpressing Flag-TH, which is further demonstrated by the CCK8 assays (Fig. 3E). Consistent with that, knock-down of TH in HCC cells were found improves the proliferation rate of HCC cells (Fig. 3F).

### TH interacts with Smad2 in a TGFβ1-dependent manner

**Fig. 4:**
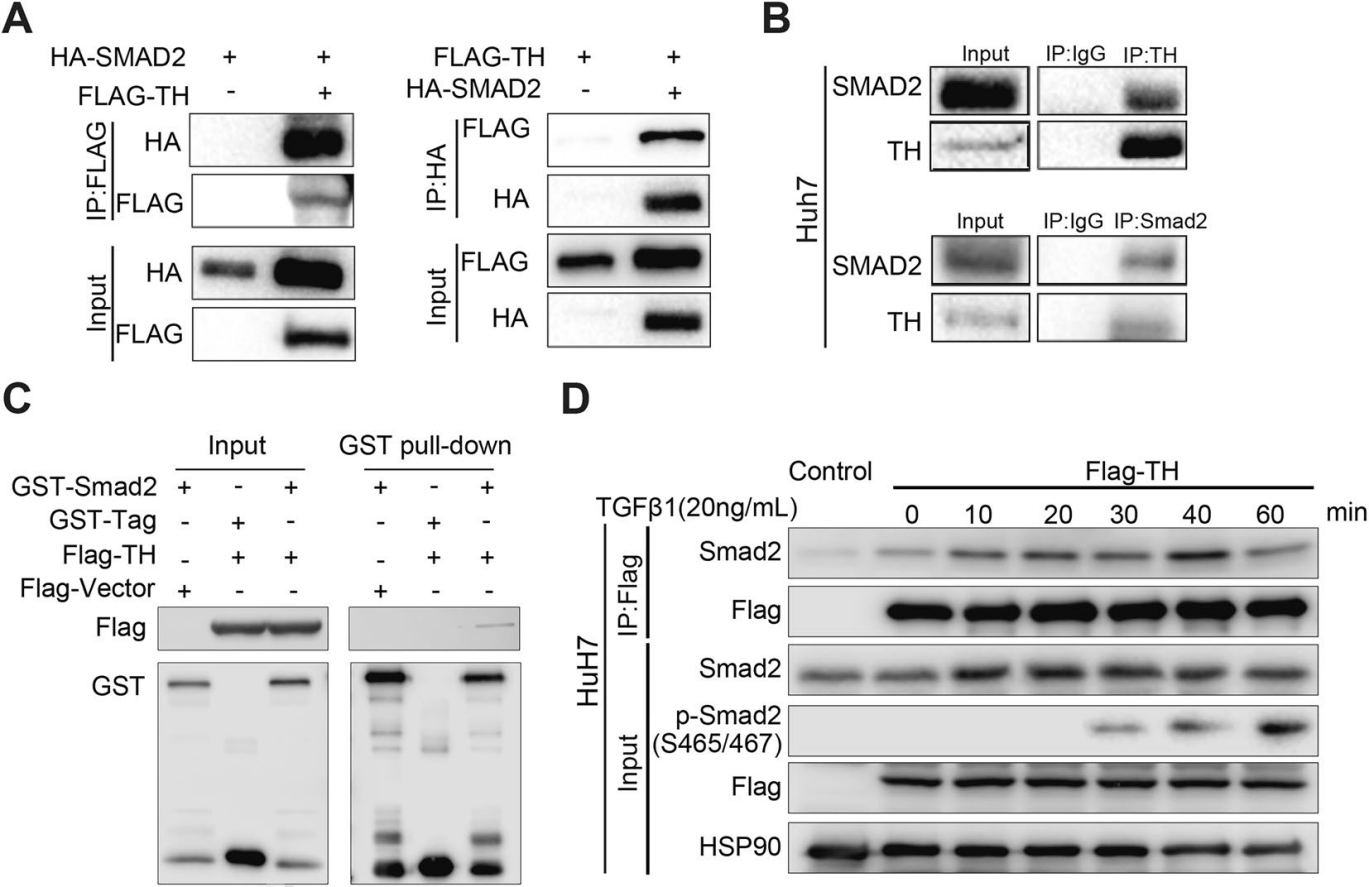
TH interacts with Smad2 in a TGFβ1-dependent manner. **A** Co-immunoprecipitation was conducted to identify the interaction involving exogenously expressed Flag-TH and HA–Smad2 in HEK293T cells. **B** Endogenously interaction between TH and Smad2 in Huh7 cells was detected by performing Co-immunoprecipitation. **C** GST pull-down assay was conducted to identify the directly binding of the GST–Smad2 fusion protein and the exogenously expressed Flag-TH. **D** Co-immunoprecipitation was applied to measuring the impact of TGFβ1 treatment on the interaction between exogenously expressed Flag-TH and endogenously expressed Smad2 in Huh7 cell lines

Previous researches have demonstrated that TGFβ signaling plays promotive impact on the migration and invasion of HCC cells, we further examine the influence of TH on the activation of the TGFβ/Smad pathway. Co-IP confirmed the interaction of TH and Smad2 (Fig. 4A), which is an important R-Smad of the TGFβ/Smad pathway. Endogenous IP performed on HCC cells further confirmed the interaction of endogenous TH and Smad proteins (Fig. 4B). Directly binding between the GST–Smad2 fusion protein and TH was validated by performing the GST pull-down assay (Fig. 4C). Furthermore, treating Huh7 cells with TGFβ1 uncovered that Flag-TH interacts with Smad2 in a TGFβ1-enhanced manner (Fig. 4D). Collectively, our research demonstrated that TH interacts with Smad2.

### TH suppresses the activation of the TGFβ/Smad pathway

**Fig. 5:**
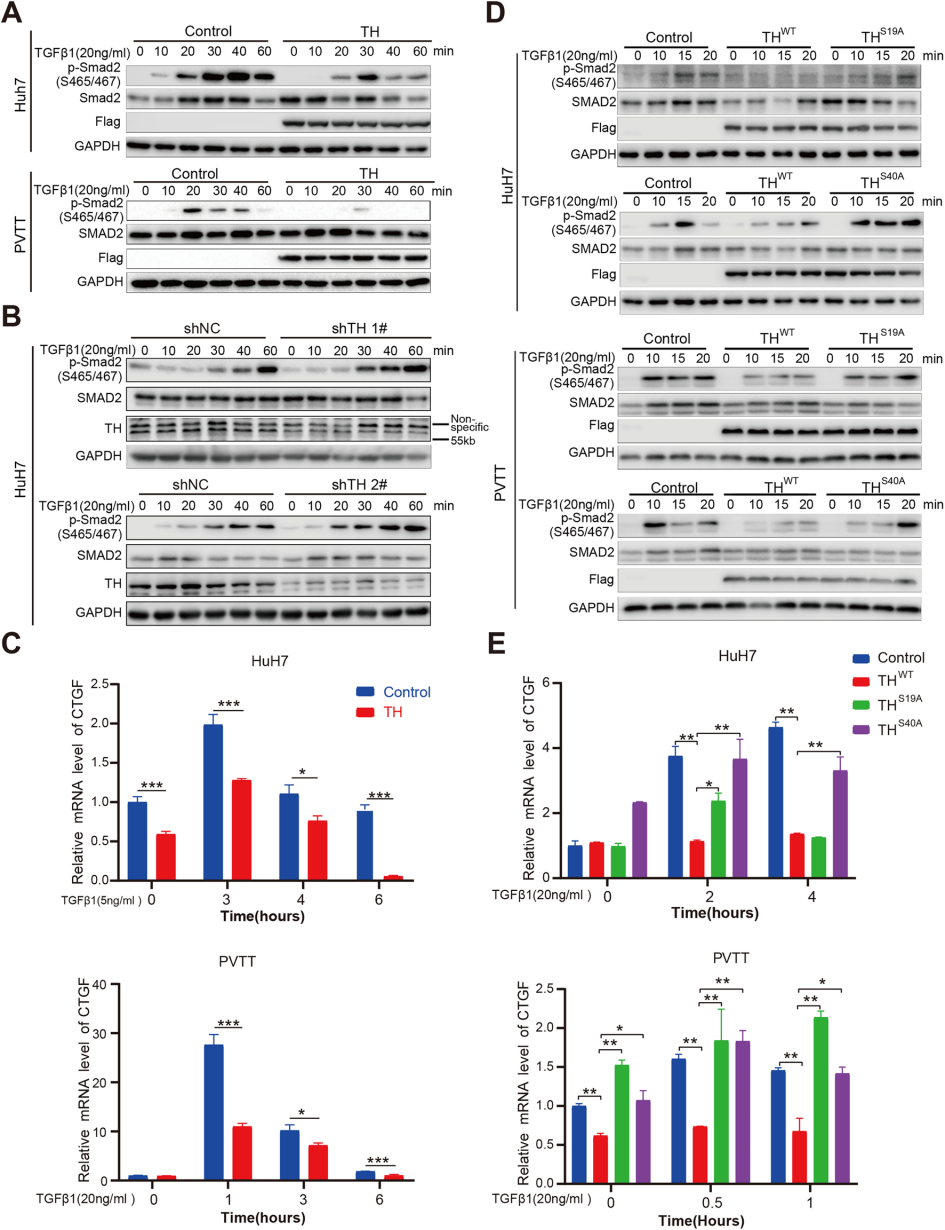
TH suppresses the activation of TGFβ/Smad signaling pathway. **A** Phosphorylation level of Smad2 (S465/467) in Huh7 and PVTT cells treated with TGFβ1 was measured by utilizing Western blotting to evaluate the impact of TH overexpression. **B** Phosphorylation level of Smad2 (S465/467) in Huh7 cells with TH knockdown treated with TGFβ1 was evaluated by western blotting. **C** qPCR was conducted to identify the impact of TH overexpressed in Huh7 and PVTT cell lines on the translational induction of CTGF mRNA in response to TGFβ1 treatment. *P < 0.05; ***P < 0.001. **D** Western blotting was conducted to evaluate the impact of TH^WT^, TH^S19A^ and TH^S40A^ on the phosphorylation of Smad2 (S465/467) in Huh7 and PVTT cells. **E** qPCR was applied to identify the impact of TH^WT^, TH^S19A^ and TH.^S40A^ overexpression on the mRNA levels of CTGF after treating Huh7 and PVTT cell lines with TGF-β1. *P < 0.05; **P < 0.01; ***P < 0.001

The interaction between TH and Smad2 prompted us to examine whether TH suppressed the activation of TGFβ/Smad signaling. Overexpression of TH inhibited the induction of p-Smad2 (Ser465/467) in both Huh7 and PVTT cells when treating the cell lines with TGFβ1 (Fig. 5A). Furthermore, knockdown of TH greatly enhanced the activation of TGFβ/Smad signaling in HCC cells (Fig. 5B). CTGF is the target gene of TGFβ/Smad signaling, and the mRNA level of CTGF reflects the transcriptional activity of TGFβ/Smad signaling [20]. Overexpression of TH impaired the induction of CTGF mRNA by TGFβ1 in HCC cells (Fig. 5C).

The inhibitory impact of wild-type TH on the activation of p-Smad2 (Ser465/467) was eliminated when the amino acids S19 and S40 mutated to alanine in HCC cell lines(A) (Fig. 5D). Consistently, the mutant forms of TH (S19A and S40A) successfully reversed the suppression of CTGF mRNA when treating Huh7 and PVTT cell lines with TGFβ1 (Fig. 5E). This provides further evidence that the TH-mediated inhibition of the TGFβ/Smad pathway depends on the phosphorylation of S19 and S40.

Phosphorylation of TH at serine residues 19 and 40 is crucial for the stability and activity of the TH protein [16]. The Mutation of S19 and S40 to alanine (A) individually or together abolished the inhibitory effect on the activation of TGFβ/Smad signaling of wild-type TH in HCC cells (Fig. 5D). Consistently, mutant TH (S19A and S40A) rescued the inhibition of CTGF mRNA upon TGFβ1 treatment in Huh7 and PVTT cells (Fig. 5E), further conforming the necessity of the phosphorylation of S19 and S40 of TH on mediating the inhibition of the TGFβ/Smad pathway. To summarize, TH suppresses TGFβ/Smad signaling pathway in HCC cells Additional file 1.

### Phosphorylation at the serine residues S19 and S40 of TH is crucial for its inhibitory functions in HCC cells

**Fig. 6:**
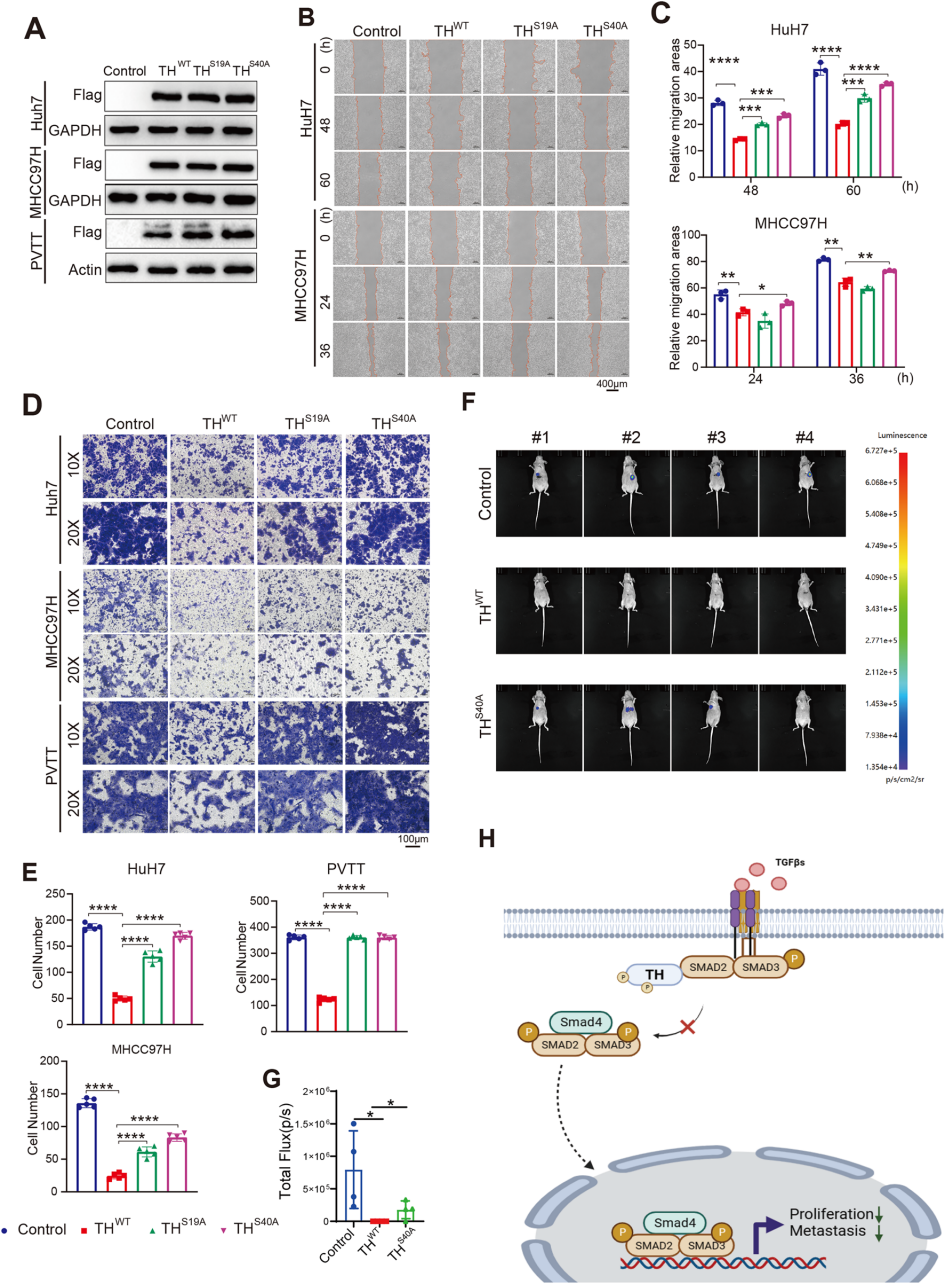
Phosphorylation of TH is crucial for its inhibitory effects on the motility of HCC cells. **A** Flag-TH^WT^, Flag-TH^S19A^ and Flag-TH^S40A^ were overexpressed in Huh7, MHCC97H, as well as PVTT cells, and the expression was detected by Western blotting. **B**, **C** Wound-healing assay was conducted to identify the impact of TH^WT^, TH^S19A^, and TH^S40A^ overexpression on the migration of Huh7 as well as MHCC97H cell lines (**B**). The migratory areas were measured and analyzed (**C**). *P < 0.05; **P < 0.01; ***P < 0.001; ****P < 0.0001. **D**, **E** Transwell invasion assays were performed to evaluate the impact of TH^WT^, TH^S19A^, and TH^S40A^ overexpression on the invasion of Huh7, MHCC97H, as well as PVTT cells. The number of invasive cells was subsequently counted and analyzed. ****P < 0.0001. **F**, **G** Metastatic Huh7 cells labeled with luciferase lentivirus (Huh7–Luci) and overexpressing TH^WT^ and TH^S40A^ was examined. The bioluminescence values were measured and analyzed 4 weeks after inject cells into the mice through the tail vein by intraperitoneal injection of D-fluorescein potassium. *P < 0.05. **H** Graphic abstract. TH inhibited development of HCC cells by interacting with Smad2 and suppressing TGFβ signaling activation

Subsequently, we examined the effect of phosphorylation at serine residues S19 and S40 for the inhibitory impacts of TH on the migration and invasion of HCC cells. We established HCC cell lines (Huh7, MHCC97H, as well as PVTT) overexpressing the wild-type TH and mutant TH (S19A and S40A) (Fig. 6A). The wound-healing assay demonstrated that the S19A and S40A mutations of TH successfully rescued the migratory capacity of HCC cells (Huh7 and MHCC97H) in comparison to the wild-type TH (Fig. 6B, C). Consistently, transwell invasion assays demonstrated that the S19A as well as S40A mutations rescued the invasion of HCC cells (Huh7 as well as MHCC97H) in comparison to the wild-type TH (Fig. 6D, E).

Given that TH suppresses HCC development, especially on the migration and invasion ability in vitro, we proceeded to identify whether TH exhibiting an inhibitory effect on the metastasis in vivo. Huh7 cells were infected with lentivirus to stably express luciferase, so that luciferase activity served as an indicator of tumor cells involved in metastasis. Subsequently, we overexpressed wild-type TH and the S40A mutant in Huh7–Luci cells and then injected into the tail veins of BALB/c nude mice. Bioluminescence imaging was conducted after a period of 4 weeks. The overexpression of wild-type TH significantly suppressed the metastasis of Huh7 cells in comparison to the control group (Fig. 6F, G). However, the S40A mutation partially eliminated the inhibitory effect of wild-type TH on metastasis (Fig. 6F, G).

## Discussion

Hepatocellular carcinoma (HCC), the predominant primary liver malignancy, as the major contributor to cancer-related mortality and an unfavorable prognosis [3, 21]. Metastasis remains the majority factor of cancer-related mortality in patients diagnosed with HCC [3]. This research uncovered that the expression of TH in HCC tissues was down-regulated and associated with clinical characteristics. Furthermore, TH inhibited the proliferation and metastasis of HCC cells by suppressing TGFβ signaling (Fig. 6H). These findings suggest that TH is potential to be a biomarker and serve as a therapeutic target for metastatic HCC.

Our research revealed a novel non-metabolic function of TH. Tyrosine hydroxylase facilitates the process of hydroxylating L-tyrosine (L-Tyr) and therefore generate dopamine (DA). When TH was found dysfunction, and therefore result in the deficiency of the DA synthesis, which ultimately leads to Parkinsonism [15, 22]. In spite of the crucial roles of TH in protecting against Parkinson’s disease, mere research had conduct to explore its role in malignancies. Over past decades, numerous metabolic enzymes have been discovered the novel non-metabolic roles [11, 14]. The non-metabolic function of TH remains to be uncovering. This study demonstrated that TH can directly bind the Smad2 protein to inhibit the activation of the TGFβ/Smad pathway through nonmetabolic functions, further uncovering the nonmetabolic functions of TH in the progression of HCC and broadening the knowledge about TH. Recently, the modification of metabolic enzymes has attracted much interest. Phosphorylation of TH at serine residues 19, 31, and 40 [16], which regulates its activity and stability, has been investigated thoroughly. Numerous studies have been performed to identify the kinases for the phosphorylation of S40 in TH [21], and eight different protein kinases were found [23–26]. TH has been found to exist as a tetramer, composed of a dimer of dimers [27]. In vitro assays have demonstrated that catecholamine binding to the dimers of TH inhibits its activity [28]. It has been elucidated that both subunits on each dimer of TH, when phosphorylated at Ser40 by PKA, are able to release catecholamine and fully restore the activity of TH [28]. In addition, CaMPKII and p38 have been reported to phosphorylate S19 in TH [24, 29]. Phosphorylated TH differs from the wild-type TH, as an in vitro study has revealed that the protein 14-3-3 specifically binds to phosphorylated TH at either Ser19 or Ser40. Furthermore, this phosphorylation event enhances the affinity between TH and the 14-3-3 protein [30]. In this study, we showed that phosphorylation of S19 and S40 are vital for the function of TH in downregulating the TGFβ/Smad pathway and suppressing the invasion and metastasis ability of HCC cells, which stressed the biological significance of the phosphorylation of TH.

Dysregulation of TGF-β signaling contributes to the progression of several haptic illnesses, besides this, has a predominant role in tumor progression of HCC through mediating EMT progression so that promoting cancer cell metastasis [21, 31]. Furthermore, the abnormal activation of TGF-β signaling in HCC leads to an immunosuppressive tumor microenvironment, mostly by suppressing the activity of effector T cells [18]. For instance, TGF-β suppresses the activity of natural killer cells and enhances the population of regulatory T cells [32, 33]. TGF-β also enhances the process of antigen-specific T-cell exhaustion by increasing the expression of PD-1 [34]. In consistent with this, combinational usage of TGF-β inhibition and ICBs is presently being evaluated in clinical studies at an early stage. Patients with little activation of TGF-β signaling had a more favorable result compared to those with either activated or inactivated TGF-β signaling. Our research provides evidence that TH suppresses the cascade activations of TGFβ/Smad pathway. However, more research remains required to determine whether down-regulation of the TGFβ/Smad pathway by TH contributes to the immunosuppressive tumor micro-environment in HCC and thus facilitates the metastasis of HCC cells.

To summarize, our study demonstrates that TH hinders the proliferation and metastasis of HCC cells by impeding the cascade activation of TGFβ/Smad signaling through a phosphorylation-dependent manner. These results suggest that TH could function as both a biomarker and a therapeutic target for HCC.

## Conclusions

To summarize, our investigation revealed the non-metabolic function of TH in HCC. Decreased expression of TH in HCC patients shown positive association with survival and negatively correlated with tumor size, tumor number, AFP level and cirrhosis. Moreover, functional experiments found that TH inhibited proliferation, migration, invasion, as well as metastasis of HCC cells. TH binds with Smad2 and impairs the activation of TGFβ/Smad signaling in a phosphorylation-dependent manner. Collectively, our study propose that TH may act as both a biomarker and therapeutic target in HCC treatment.

### Supplementary Information

**Additional file 1: **All row immuno-blot data of this article are present on this figure.

## Data Availability

All data and materials presented in this study are available in this article.

## Abbreviations

HCC: Hepatocellular carcinoma
TH: Tyrosine hydroxylase
AAAH: Aromatic amino acid hydroxylase
CAs: Catecholamines
DA: Dopamine
BH4: Tetrahydrobiopterin
L-Tyr: L-tyrosine
AFP: Alpha-fetoprotein
CTGF: Connective tissue growth factor
EMT: Epithelial–mesenchymal transition
L-DOPA: L-3,4-dihydroxyphenylalanine
DAB: 3,3ʹ-Diaminobenzidine
CaMPKII: Calcium calmodulin dependent protein kinase II
TME: Tumor microenvironment
PD-1: Programmed cell death protein 1
