# Supplementary figures and images for: Tyrosine hydroxylase inhibits HCC progression by downregulating TGFβ/Smad signaling
Source: Eur J Med Res. 2024 Apr 12;29:228. doi: 10.1186/s40001-024-01703-z (PMC11015545; doi:10.1186/s40001-024-01703-z)

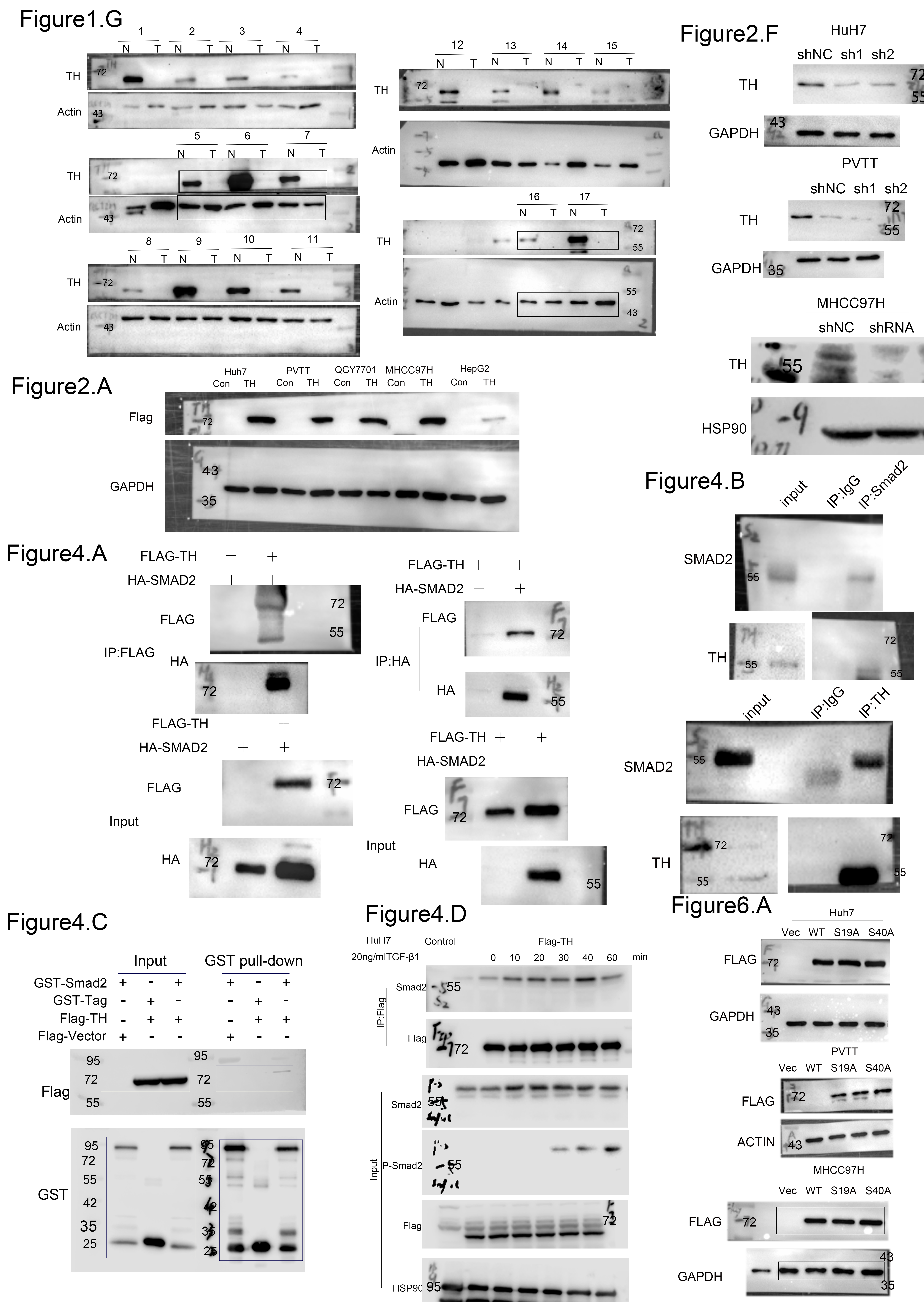

Supplement: Supplementary file 1 — Additional file 1: All row immuno-blot data of this article are present on this figure. [file 40001_2024_1703_MOESM1_ESM.zip › New folder/Row data -1 .tif]

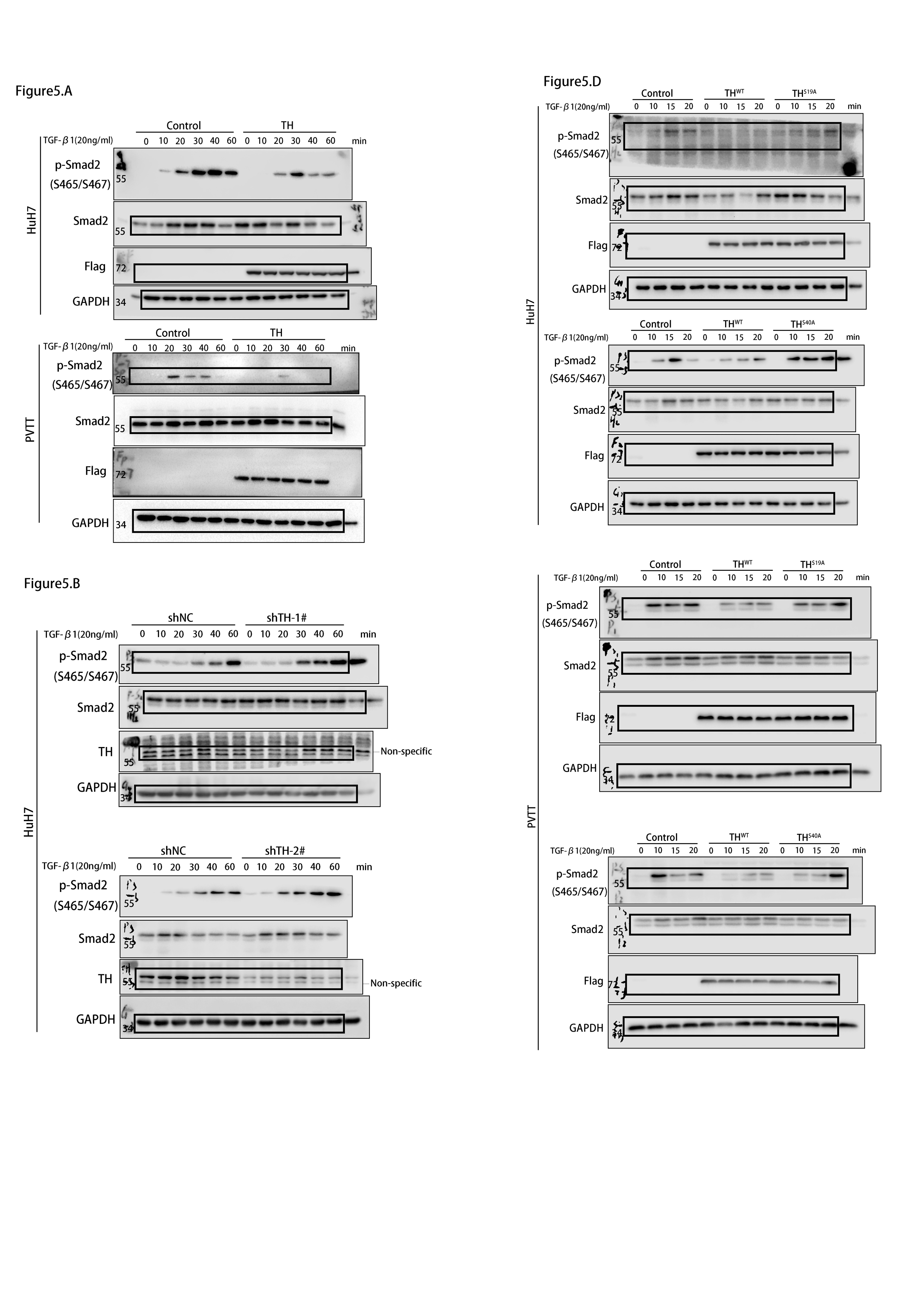

Supplement: Supplementary file 1 — Additional file 1: All row immuno-blot data of this article are present on this figure. [file 40001_2024_1703_MOESM1_ESM.zip › New folder/Row data-2.tif]
